# quareticctivity of *Solidago canadensis*cultivated ingypt and etermination of theostioactiveraction

**DOI:** 10.22037/ijpr.2019.2390

**Published:** 2019

**Authors:** Passent Mahmoud Abdel Baki, Moshera Mohamed El-Sherei, Amal Elsayed Khaleel, Amira Ahmed Abdel Motaal, Heba Mohammed Ibrahim Abdallah

**Affiliations:** a *Department of Pharmacognosy, Faculty of Pharmacy, Cairo University, Kasr El-Aini St., Cairo 11562, Egypt. *; b *Department of Pharmacognosy, College of Pharmacy, King Khalid University, Abha, Kingdom of Saudi Arabia. *; c *Department of Pharmacology, Medical Research Division, National Research Center, Giza 12622, Egypt.*

**Keywords:** Aquaretic activity, Flavonoids, Kaempferol-3-O-β-ᴅ-apiofuranoside, Phenolics, Saponins, Thymidine

## Abstract

Despite the traditional use of *Solidago canadensis *L. (*Asteraceae*) as a diuretic drug, there is a scarcity in scientific data concerning the activity of its different extracts and fractions as well as the class of constituents responsible for this diuretic action. A comparative study was carried out for the diuretic activities of the different standardized extracts and fractions of the flowering aerial parts of *S. canadensis, *as well as isolation of compounds from the most biologically active fraction. The ethanol extract and its ethyl acetate fraction (EA) showed the highest aquaretic activities (91 and 58% at a dose of 400 mg/Kg b.wt., respectively) compared to 100% of furosemide at 20 mg/Kg b.wt.. Their activities were higher than Cystinol® and spironolactone reference standards (74% and 59% of furosemide, respectively). EA showed the highest total phenolic and flavonoid contents among the tested fractions of the ethanol and aqueous extracts (9.38 ± 0.004 g GAE and 39.75 ± 0.005 g RE/100 g dried extract, respectively). Eight flavonoids, 2 phenolic acids and 1 nucleoside were isolated from EA. This is the first report of a comparative study between the aquaretic activities of the different extracts, fractions and essential oil of *S. canadensis*, as well as isolation of thyimidine **(1)**, isorhamnetin-3-*O*-*β*-ᴅ-glucopyranoside (**2**), kaempferol-3-*O*-(6”-*O*-acetyl)-*β*-ᴅ-glucopyranoside (**4**), quercetin-3-*O*-(6”-*O*-acetyl)-*β*-ᴅ-glucopyranoside (**5**), and kaempferol-3-*O*-*β*-ᴅ-apiofuranoside (**7**) from genus *Solidago*.

## Introduction

Phytopharmaceuticals were successfully used in the therapy of the urinary tract with parallel administration of synthetic drugs especially those used as diuretics (1). Diuretics are commonly defined as drugs that promote the rate of urine flow by the kidneys (2). The commonly used synthetic diuretics (*viz* thiazides and furosemide) have been associated with many side effects such as disturbances of electrolytes, acid-base and water balance, changes in uric-acid, carbohydrate and lipid metabolism, and drug interactions (3). Therefore, herbal diuretics could be considered as a better therapeutic option, having relatively safer and milder actions, compared to synthetic diuretics which cause several adverse effects due to their strong saluretic actions (4). Numerous herbs were traditionally considered as diuretics. Among those herbs are members of genus *Solidago *belonging to family *Asteraceae* (5).

Numerous interesting secondary metabolites such as flavonoids, triterpenoids, saponins, phenolic acids, glucosides, polysaccharides, diterpenes, and essential oils were reported in genus *Solidago *(6). Most commonly used in phytotherapy are the apical shoots of goldenrod, known as *Herba Solidaginis* (7). *Herba Solidaginis* were applied in the middle ages in nephrolithiasis, urinary tract, and prostate diseases, while the flowers and leaves were used as natural yellow dyes (6). Goldenrods were forgotten for a while, but found its place again in modern phytotherapy and their demand has been rising over the past few years (6, 8). Several species of *Solidago* were reported to exhibit diuretic, spasmolytic, cytotoxic, anti-microbial, anti-mutagenic, anti-inflammatory, immunobiological, gastroprotective, ulcer-healing, amoebicidal and amoebistatic activities (9-21). The essential oils of different goldenrods were reported to possess anti-microbial activity (9-14, 16-22).

*Solidago canadensis *L., also known as Canadian goldenrod, is native to North America (23, 24). It is an erect perennial herb that is widely used as a landscape flowering plant, and in cut flower arrangements and bouquets (8). Canadian goldenrod has been used in folk medicine for centuries as urological and antiphlogistical therapy, febrifuge, analgesic, gastro-intestinal tract and liver aids, and in burns and ulcer treatment (25-30). It was reported to exhibit several biological activities including diuretic, anti-microbial, cytotoxic, antioxidant, inhibiting activity to the lyase of DNA polymerase, antimutagenic activities (9, 16, 24, 31-33). Its essential oil was reported to possess cytotoxic and anti-microbial activities (23, 34-36). Earlier investigations on the plant led to the isolation of flavonoids, phenolic acids, saponins, alkaloids, polyacetylenes, terpenes and sterols (31, 33-45).

In the past few years, the extract of the flowering aerial parts of *Solidago virgaurea* L. was launched in the Egyptian market under the trade name of Cystinol® at a dose of 400 mg. It is used for the treatment of urolithiasis by promoting the excretion of water more than the electrolytes and increasing renal blood flow. This facilitates the washout of bacteria from the urinary tract, prevents crystal formation, and hence kidney stones (46). 

Having Cystinol® in the Egyptian market, it was found interesting to investigate the diuretic activity of the essential oil and crude extracts of the flowering aerial parts of *S. canadensis *cultivated in Egypt using different solvents for extraction (70% ethanol and water). A biologically guided fractionation of the crude extracts was carried out to determine the most active fraction and its content of active constituents.

## Experimental

*Plant material*

Samples of *Solidago canadensis* L. were collected during the years 2010-2013 from El-Mansoureya, Giza, Egypt. The plant was kindly authenticated by the temperate regional team of the Royal Botanical Gardens, Kew., London, U.K. Voucher specimen of the plant (24.02.2013) was deposited at the herbarium of the Pharmacognosy Department, Faculty of Pharmacy, Cairo University. 

*Plant extraction*

*A- Ethanolic extract*

The air-dried powdered flowering aerial parts of *S. canadensis *(1 kg) was exhaustively extracted with 70% ethanol by cold maceration to give 230 g extract. An aliquot of the dry residue (200 g) was subjected to liquid-liquid fractionation with *n*-hexane, methylene chloride, ethyl acetate and *n*-butanol saturated with water and concentrated to give 10.4, 6.8, 17.2 and 22 g of dried fractions, respectively. The remaining water of the ethanol extract was lyophilized and weighed (122 g). 

*B- Aqueous extract*

The air-dried powdered flowering aerial parts (1 kg) were macerated in boiling distilled water for 20 min. The aqueous extract was lyophilized to give 206 g residue. An aliquot of the lyophilized residue (200 g) was extracted with methylene chloride, ethyl acetate and *n*-butanol saturated with water to give 12, 18, and 26 g of the dried fractions, respectively. The remaining water left after fractionation was lyophilized and weighed (138 g). The extracts and fractions were stored at 5 °C for both the phytochemical and biological investigations.

*C- Preparation of essential oil*

The fresh flowering aerial parts (200 g) were subjected to hydrodistillation using a Clavenger′s apparatus according to the procedures described in the Egyptian Pharmacopœia (47). The obtained essential oil sample wasdried over anhydrous sodium sulphate. The hydrodistilled oil was saved in a refrigerator (4 ºC) in a tightly sealed container.

*Chemicals and equipment*

Cystinol® was purchased from Atos Pharma (Cairo, Egypt). Furosemide and spironolactonewere obtained from Sigma-Aldrich (Darmstadt, Germany) and used as reference diuretic drugs.

The total phenolic content was estimated using Folin-Ciocalteu′s colourimetric assay, while the total flavonoids were determined using the AlCl_3_colourimetric assay (48, 49). The total saponin contentwas carried out using the vanillin colourimetric assay (50). Authentics as gallic acid, rutin and ursolic acid were obtained from E-Merck, Darmstadt, Germany. Phenolics and sugars used as reference standards in co-chromatography (PC and TLC) were purchased from Sigma Chemicals Co. (St. Louis, MO, USA). Diaion HP-20 AG (75-150 µ, Sigma-Aldrich Chemicals, Germany), silica gel 60, and silica gel RP-18 (70-230 mesh, Fluka, Sigma-Aldrich Chemicals, Germany), silica gel 60 (35-70 mesh, ASTM Germany) and sephadex LH-20 (Pharmacia Fine Chemicals AB, Uppsala, Sweden) were used for column chromatography (CC). Thin layer chromatography (TLC) was performed on silica gel 60 F_254_ and silica gel RP-18 (Fluka, Sigma-Aldrich Chemicals, Germany) using the following solvent systems: S_1_, methylene chloride-methanol-formic acid (95:5:0.2 v/v/v); S_2_, methylene chloride-methanol-formic acid (90:10:0.2 v/v/v); S_3_, methylene chloride-methanol-formic acid (85:15:0.2 v/v/v); S_4_, ethyl acetate-methanol-water (100:16:13 v/v/v); S_5_, ethyl acetate-formic acid-glacial acetic acid-water (100:11:11:10 v/v/v/v). The chromatograms were visualized under UV light (at 254 and 366 nm) before and after exposure to ammonia vapour and spraying with AlCl_3_, FeCl_3 _as well as after spraying with natural product-polyethylene glycol (NP/PEG) and *p*-anisaldehyde spray reagents. Paper chromatography was conducted on Whatmann No. 1 filter paper (Whatmann, Ltd., Maidstone, Kent, England) using solvent system S_6_, *n*-butanol-acetic acid-water (4:1:2 v/v/v, upper phase) and visualized by spraying with aniline phthalate spray reagent. Shift reagents for UV spectroscopy according to the published procedures and chemicals used were obtained from E-Merck, Darmstadt, Germany (51). Melting points (uncorrected) were determined on anelectrothermal 9100 (UK). UV spectra were recorded in a Jenway model 6800 spectrophotometer. ^1^H-NMR (300, 400, 600 MHz) and ^13^C-NMR (75, 100, 150 MHz) were measured on a Varian Mercury NMR-spectrometer (Japan), Bruker Ascend TM 400/R NMR spectrometer and Bruker Ascend TM 600/R NMR spectrometer, respectively. The NMR spectra were recorded in CD_3_OD and DMSO-d6 and chemical shifts were given in δ (ppm) relative to TMS as an internal standard. EI-MS was performed on Varian Mat 711, Finnigan SS Q 7000.

*Evaluation of the pharmacological activity*

*Animals*

Adult male albino rats of Wistar strain (120-150 g), obtained from the animal house colony at the National Research Center (Dokki, Giza, Egypt), were utilized for determination of the LD_50_ and assessment of the diuretic activity. They were housed in steel cages at standardized conditions of temperature and humidity and fed with standard pellets and water *ad libitum*. All experimental procedures were conducted in accordance with the internally accepted principles for laboratory animal use and care, and were approved by the Ethics Committee No. MP (4) in accordance with recommendations for the proper care and use of laboratory animals (NIH Publication No. 85-23; revised 1985).

*Determination of median lethal dose (LD*_50_ *)*

The LD_50 _of both the ethanol and aqueous extracts was determined following Karber′s procedure (1931) (52). Five groups, each of six rats, received both plant extracts separately in doses ranging from 1 to 4 g/kg b.wt. The LD_50_ of the tested extracts was calculated according to the following formula:

 LD_50_ =Dm -∑(z x d)n

Where:

Dm = The largest dose that killed all animals.

z = Mean of dead animals between 2 successive groups.

d = The constant factor between 2 successive doses.

n = Number of animals in each group.

Σ = The sum of (z × d).

*Evaluation of the diuretic activity*

The 70% ethanol and aqueous extracts, their fractions, and the essential oil of the flowering aerial parts were tested for their diuretic activities as well as their effect on the excretion of potassium and sodium in urine according to the method of Lipschitz *et al.* 1943 (53). Three diuretic drugs namely furosemide (20 mg/kg b.wt.), spironolactone (25 mg/kg b.wt.) and Cystinol® (400 mg/kg/b.wt.) were used as reference standards. Oral doses of 200 and 400 mg/kg b.wt.of the tested *Solidago* extracts and fractions were selected for the study, based on the marketed dose of Cystinol®. The rats were fasted and deprived of water for 18 h before the experiment. They were divided into 28 groups of six animals each. The rats of each group were subjected to the specified treatment and the control group received 1 mL 0.9% NaCl/100 g b.wt. The following parameters were estimated:

*Urine output*

Immediately after the treatment, the animals were individually placed in metabolic cages specially designed to separate urine and faeces. During this period, neither food nor water was made available to the animals. The room temperature was maintained at 27-29 °C. The urine was collected in measuring cylinders up to 24 h after treatment for all control and treated groups. Urine volume was expressed as mL/kg (54). 

Diuretic action:Urinary excretion of test group Urinary excretion of control group (55, 56).

Diuretic activity: $\frac{\mathrm{Diuretic action of extract}}{\mathrm{Diuretic action of standard}}$x 100 (55).

*Estimation of electrolytes*

The electrolyte (Na^+^, K^+^) content was estimated in the urine using the commercially available kit (Biodiagnostic Co., Giza, Egypt).

Na^+^/ K ^+^ratio:Concentration of Na+in urineConcentration of K+in urine.

*Statistical analysis*

The data obtained were presented as mean ± standard error (SE) and statistically analyzed using ANOVA followed by LSD post-hoc test. The values were determined to be significant when *p-*value was less than 0.05 (*p *< 0.05).

*Isolation of the components of the ethylacetate fraction of the 70% ethanolextract (EA)*

EA (16 g) was chromatographed on a Diaion column (35 cm L × 3.5 cm D). Gradient elution with water/methanol mixtures was adopted. Fractions, 200 mL each, were collected and monitored by TLC using solvent system S_2_. Similar fractions were pooled together and the solvents were separately evaporated under reduced pressure yielding three major fractions (I-III). Fraction I (0.8 g, eluted with 25% methanol in water) was rechromatographed over a silica gel 60 column (30 × 1 cm) eluted with methylene chloride: methanol (95:5 v/v) yielding compound **1 **(30 mg). Fraction II (6.5 g, eluted with 50% methanol in water) was rechromatographed over a sephadex LH 20 column (30 × 3 cm) using methylene chloride: methanol (1:1 v/v) as eluent to give three fractions (IIa, IIb and IIc). Upon evaporation of fractions IIa and IIb, two pure compounds were obtained: compound **2** (25 mg) and compound **3** (20 mg). Fraction IIc (5 g) was rechromatographed over a silica gel 60 column (25 × 3 cm). Elution with methylene chloride: methanol (95:5 v/v) was adopted yielding four fractions IIc_1-4_. Upon evaporation, IIc_1_ yielded compound **4** (45 mg) while, IIc_2_ yielded compound **5** (40 mg). FractionIIc_3_ (200 mg) was rechromatographed on RP-18 column (10 × 1 cm), eluted with H_2_O: MeOH (80:20 v/v) to yield of compound **6** (45 mg). FractionIIc_4_ (2.8 g) was rechromatographed over sephadex LH20 column (20 × 1.5 cm) using methanol: water (1:1 v/v) as eluent yielding two main fractions (IIc_4_i and IIc_4_ii). IIc_4_i gave compound **7** (20 mg). IIc_4_ii (1g) was rechromatographed over a silica gel 60 column (13 × 1 cm) and eluted by methylene chloride: methanol (95: 5 v/v), yielding two compounds, compound **8 **(45 mg) and compound **9 **(20 mg). Fraction III (1.8 g, eluted with 75 and 100% methanol in water) was rechromatographed over a sephadex LH 20 column (30 × 3 cm). Elution with methanol: H_2_O (90:10 v/v) was adopted giving two fractions IIIa and IIIb. Fraction IIIa yielded compound **10** (40 mg). Similarly, IIIb (83 mg) was rechromatographed over a sephadex LH 20 column (13 × 1 cm). Elution with methanol: water (1:1 v/v) yielded compound **11** (40 mg).

*Spectroscopic data of the isolated compounds*

*Compound* ***1*** (*thymidine*)

EI/MS (70 eV rel. int.), m/z at 126 [M–deoxyribose]^+^, (32.22%); 83 [M-deoxyribose – HNCO]^+^, (25.88%); 70 [M^+ ^– deoxyribose– C_3_H_4_O]^+^, (31.53%); 55 [M –deoxyribose– HNCO –CO]^+^, (100%, base peak); 54 [M–deoxyribose–HNCO – CO –H]^+^, (76.10%); 28 [M –deoxyribose– HNCO – CO – H – C_2_H_2_]^+^, (89%); 27 [M –deoxyribose– HNCO – CO – H – C_2_H_2 _– H]^+^, (34.43%).

**Figure 1:**
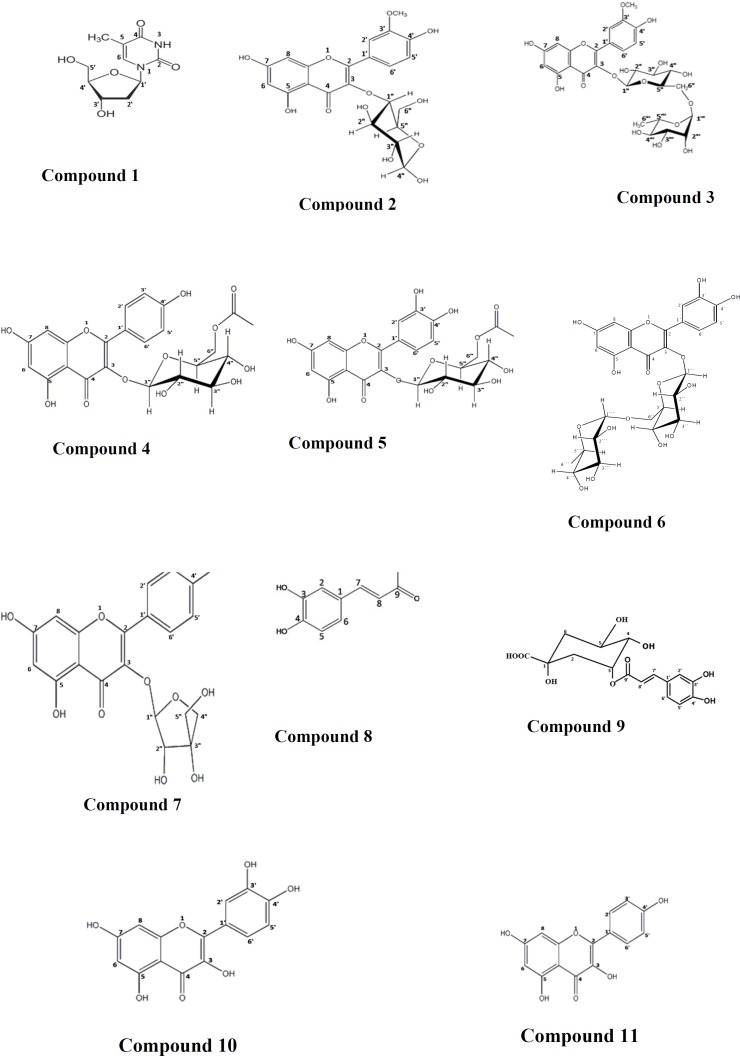
Structures of the isolated compounds

Structures of the isolated compounds are shown in Figure 1 and their spectral data were recorded in Tables 1-4.

## Results

*Determination of median lethal dose*

Both the ethanol and aqueous extracts were safe and non-toxic under the present experimental conditions up to 4 g/kg b.wt. The extracts are considered safe in the range of the administered doses (57).

*Effect on urine volume*

The reference diuretic drugs furosemide, spironolactone, and Cystinol® significantly increased the urine output when compared to the control group (Table 5). The diuretic activities of the tested samples were calculated relative to furosemide, as it proved to be the most potent diuretic reference drug. All the tested samples showed a significant increase in the volume of urine output except the *n*-butanol and the remaining water fractions of the aqueous extract (Table 5). The 70% ethanolextract of the aerial parts (400 mg/kg/b.wt.) exhibited the highest diuretic activity of all tested samples comparable to that of furosemide (91% of furosemide activity), and higher than spironolactone and Cystinol® which exhibited 59% and 74% of furosemide activity, respectively (Table 5). The aqueous extract (400 mg/kg/b.wt.) exhibited lower diuretic activity than that of the 70% ethanol extract and all the used reference diuretics (46% of furosemide). Whereas, the ethyl acetate fraction of the 70% ethanol extract (EA) (400 mg/kg/b.wt.) showed the highest diuretic activity amongst all fractions representing 58% of furosemide activity and compared to spironolactone (potassium sparing diuretic). However, it was less potent than the parent 70% ethanol extract at the same dose level. The essential oil (400 mg/kg/b.wt.) showed poor diuretic activity (31% of furosemide activity).

*Effect on urinary electrolyte excretion*

Furosemide significantly increased the excretion of urinary electrolytes. Spironolactone (potassium sparing diuretic) increased the excretion of sodium iononly, while Cystinol® did not affect the excretion of sodium and potassium in urine (aquaretic). Administration of all the tested extracts, fractions and the essential oil of the aerial parts at both dose levels (200 and 400 mg/kg b.wt.) did not affect the urinary electrolyte excretion (Table 5). The Na^+^/K^+^ excretion ratio was uniform (1.27 to 1.36) in all the tested plant samples.

*Spectrophotometric estimation of the total phenolic, flavonoid and saponin contents*

The 70% ethanol extract of the total aerial parts showed higher total phenolic and flavonoid contents than their aqueous extract while the saponins were more concentrated in the aqueous extract. The most active EA showed the highest total phenolic and flavonoid contents amongst all the tested fractions of both extracts, whereas the *n*-butanol fraction of the aqueous extract was the highest in the saponin content (Table 6).

*Purification of the phenolic and flavonoid rich fraction (EA)*

The quantitative determination of the major constituents in the different fractions and correlating their relative concentrations to their diuretic activities, revealed that the total phenolics and flavonoids may be responsible for this activity. EA was subjected to the purification and isolationof its major constituents as it possessed the highest diuretic activity and was standardized to contain the highest amount of total phenolics and flavonoidsamong other fractions. Thymidine **(1)**, isorhamnetin-3-*O*-*β*-ᴅ-glucopyranoside **(2)**, narcissin **(3)**, kaempferol-3-*O*-(6”-*O*-acetyl)-*β*-ᴅ-glucopyranoside **(4)**, quercetin-3-*O*-(6”-*O*-acetyl)-*β*-ᴅ-glucopyranoside **(5)**, rutin **(6)**, kaempferol-3-*O*-*β*-ᴅ-apiofuranoside **(7)**, caffeic acid **(8)**, neochlorogenic acid **(9)**, quercetin **(10),** and kaempferol **(11)** were isolated from the ethyl acetate fraction (Figure 1). Identification of the isolated compounds was achieved by their physicochemical and spectral data, and by comparison with the available authentic samples and with the published data (51, 58-67).

## Discussion

Preliminary phytochemical screening of the air-dried flowering aerial parts of *S. canadensis *deduced that the main constituents of the plant were essential oils, free and combined flavonoids, and saponins. Guided by the available literature and the results of the phytochemical screening, it was found interesting to study the main active constituents of the plant and the solvent of choice for the extraction of each class of constituents knowing that the essential oil of the plant was previously investigated by our group (35). Higher yield of total phenolic compounds and flavonoids was achieved by extraction with 70% ethanol rather than water. This was in accordance with the previous reports of Apati *et al.* 2002 (39). EA showed the highest diuretic activity as well as the highest total phenolic and flavonoid contents relative to the other fractions, this made it the most proper candidate for further phytochemical investigations. Optimum extraction of saponins was achieved by water rather than 70% ethanol. The saponins were concentrated in the *n*-butanolfraction of the aqueous extract.

**Table 1:** UV-shifts of theisolated flavonoids and phenolic acids

	**MeOH**	**Na methoxide **	**AlCl**_3_	**AlCl**_3_ **/HCl **	**Na acetate **	**Na acetate/boric acid**
2 isorhamnetin-3- *O*-*β*-D-glucopyranoside	254, 348	271, 326sh, 410	268, 402	268, 363sh, 398	267, 318sh, 389	255, 346
3 Narcissi	252, 347	277, 405	279, 355sh, 407	279, 345sh, 400	278, 325sh, 382	253, 347
4 kaempferol- 3-*O*-(6"-O-acetyly)-*β*-D-glucopyranoside	268, 300sh, 350	276, 328sh, 400	268, 302sh, 350, 400	274, 300sh, 348sh, 403	274, 304, 382	272, 303, 354
5 quercetin- 3-*O*-(6"-O-acetyly)-*β*-D-glucopyranoside	257, 273sh, 365	279, 329, 410	279, 303sh, 435	276, 365sh, 408	278, 328sh, 398	267, 291sh, 390
6 Rutin	258, 300sh, 358	268, 328sh, 410	270, 306sh, 426	268, 298sh, 366, 400	264, 300sh, 382	262, 308sh, 378
7 kaempferol-3-*O*-*β*-D-apiofuranoside	268, 350	274, 324sh, 400	274, 302sh, 396	274, 300sh, 394	274, 382	258, 350
8 caffeic acid	245, 290sh, 330			No Change		
9 neochlorogenic acid	290, 326			No Change		
10 Quercetin	256, 301sh, 372	247sh, 330, 406	269, 457	267, 303sh, 352sh, 429	268, 329sh, 390	259, 386
11 kaempferol	265, 292sh, 329sh, 366	280, 322sh, 418	269, 304sh, 347, 425	269, 303sh, 350, 424	274, 303sh, 390	267, 295sh, 368

**Table 2:** 1H-NMR of the isolated phenolic acids and nucleoside

**Position**	**1 (400 MHz, DMSO) thymidine**	**8 (400 MHz, CD** **3** **OD) caffeic acid**	**9 (300 MHz, DMSO) neochlorogenic acid**
2		6.94 (d, J = 2 Hz)	
3			5.01 (d, J = 3.6 Hz)
4			3.82 (br.s)
5		6.67 (d, J = 8 Hz)	3.87 (d, J = 9 Hz)
6	7.70 (s)	6.82 (dd, J = 8, 2Hz)	
7		7.41 (d, J = 16 Hz)	
8		6.10 (d, J = 16Hz)	
1'	6.15 (t)		
2'	2.03 (2H, m)		7.02 (d, J = 1.8 Hz)
3'	4.22 (q)		
4'	3.75 (dd, J = 10.4, 3.7 Hz)		
5'	3.51 (o)		6.75 (d, J = 8.4)
6'			6.95 (dd, J = 8.1, 2 Hz)
7'			7.35 (d, J = 15.9 Hz)
8'			6.07 (d, J = 16.2 Hz)
2ax, eq			2.08 (2H, m)
6ax			1.73 (d, J = 12.9 Hz)
6eq			1.90 (dd, J = 13.8, 10.8 Hz)
CH3-5	1.78 (3H, s)		
N-H	11.27 (s)		

**Table 3:** ^1^H-NMR of the isolated flavonoids

**Position**	**2** **(300 MHz, CD**_3_ **OD)**	**3** **(300 MHz, CD3OD)**	**4** **(600 MHz, DMSO)**	**5** **(600 MHz, DMSO)**	**6** **(300 MHz, DMSO)**	**7** **(400 MHz, CD**_3_ **OD)**	**10** **(300 MHz, CD**_3_ **OD)**	**11** **(400 MHz, DMSO)**
6	6.21 (d, J = 1.8Hz)	6.20 (d, J = 2.1Hz)	6.19 (d, J = 1.8Hz)	6.15 (br.s)	6.11 (br.s)	6.09 (br.s)	6.18 (br.s)	6.19 (d, J = 2Hz)
8	6.41 (d, J = 1.5Hz)	6.39 (d, J = 1.8Hz)	6.42 (d, J = 1.8Hz)	6.36 (br.s)	6.30 (br.s)	6.31 (d, J = 1.7 Hz)	6.40 (d, J = 1.5 Hz)	6.44 (d, J = 2Hz)
2'	7.92 (d, J = 1.8 Hz)	7.92 (d, J = 2.1Hz)	7.98 (dd, J = 9, 1.8Hz)	7.49 (d, J = 2.4Hz)	7.51 (br.s)	7.99 (d, J = 8.84 Hz)	7.74 (br.s)	8.03 (d, J = 8.9Hz)
3'			6.85 (dd, J = 8.4, 1.8Hz)			6.81 (d, J = 8.9 Hz)		6.92 (d, J = 8.9Hz)
5'	6.88 (d, J = 9)	6.90 (d, J = 8.4Hz)	6.85 (dd, J = 8.4, 1.8Hz)	6.80 (d, J = 8.4Hz)	6.79 (d, J = 8.7 Hz)	6.81 (d, J = 8.9 Hz)	6.89 (d, J = 8.1 Hz)	6.92 (d, J = 8.9Hz)
6'	7.58 (dd, J = 8.4, 1.8Hz)	7.58 (dd, J = 8.4, 1.8 Hz)	7.98 (dd, J = 1.8, 9Hz)	7.50 (dd, J =1.8, 8.4Hz)	7.52 (dd, J = 2.7, 8.4 Hz)	7.99 (d, J = 8.8 Hz)	7.64 (d, J = 6 Hz)	8.03 (d, J = 8.9 Hz)
OCH_3_	3.60 (3H, s)	3.65 (3H, s)						
Sugar protons	5.22 (d, J = 7.2Hz, H-1'')	5.36 (d, J = 7.8Hz, H-1'')	5.34 (d, J = 7.8 Hz, H-1'')	5.34 (d, J = 7.2Hz, H-1'')	5.28 (d, J = 7.2Hz, H-1'')	5.01 (d, J = 3.7 Hz, H-1'')		
	3.32-3.44 (6 sugar protons)	4.52 (br.s, H-1''')	3.28 - 3.95 (6 sugar protons)	3.2- 3.4 (6 sugar protons)	4.39 ( br.s, H-1''')	4.11 (d, J = 4 Hz, H-2" )		
		3.30-3.60 (10 sugar protons)	1.73 (3H, s, COCH_3_)	1.71 (3H, s, COCH_3_)	3.17- 3.72 (10 sugar protons)	4.00 (d, J = 8 Hz, H-4a")		
		1.29 (3H, br.s, CH_3_ Rh)			0.99 (3H, d, J = 6.3Hz, CH_3_ Rh)	3.55 (d, J = 8.9 Hz, H-4b")		
						3.31 (s, H-5")		

**Table 4:** 13C-NMR of the isolated compounds

**C**	**1 (100 MHz, DMSO)**	**2 (150 MHz, DMSO)**	**5 (150 MHz, DMSO)**	**7 (100 MHz, CD3OD)**	**8 (100MHz, CD3OD)**
1		-	-	-	126.40
2	150.92	156.53	156.3	157.23	114.12
3		133.06	133.12	133.10	145.65
4	164.20	177.34	177.21	176.98	148.05
5	109.81	161.19	161.21	160.74	115.10
6	136.58	98.78	98.6	99.69	121.46
7		164.47	164.5	163.20	145.39
8		93.74	93.74	95.00	113.70
9		156.42	156.41	156.10	169.63
10		103.79	103.55	105.35	
1'	84.19	120.76	121.56	122.11	
2'	40.61	130.83	115.15	131.23	
3'	70.90	115.05	148.65	115.92	
4'	87.71	160.03	144.91	160.23	
5'	61.80	115.05	116.08	115.92	
6'		130.83	121.04	131.23	
CO acetyl		169.84	169.97	-	
CH3 of acetyl		20.17	20.12	-	
1''		101.11	101.12	107.17	
2''		74.09	74.03	77.20	
3''		76.12	76.31	78.24	
4''		69.77	69.98	73.54	
5''		73.88	74	62.31	
6''		62.74	62.83	-	
CH3-5	12.72				

**Table 5:** The diuretic action, diuretic activity, Na+, K+ levels and Na+/K+ ratios of the essential oil, extracts and fractions of the aerial parts of *Solidago canadensis *L

**Treatment**			Percent of Diuretic							Electrolytes mEq/L				
			**Urine volume (mL/24 h)**		**Diuretic action**		**activity**			Na+	K+		**Na+/K+Ratio**	
Control (1 mL saline/100 g b.wt.)			1.6* ± 0.12		1.00		14			2.54 ± 0.01	1.94 ± 0.01		1.31	
Furosemide (20 mg/kg b.wt.)			11.56* ± 0.41		7.23		100			4.25 ± 0.04	2.86 ± 0.04*		1.49	
Spironolactone (25 mg/kg b.wt.)			6.88* ± 0.40		4.30		59			3.48 ± 0.01	1.95 ± 0.02		1.78	
Cystinol® (400 mg/kg b.wt.)			8.57* ± 0.40		5.36		74			2.50 ± 0.05	1.98 ± 0.03		1.26	
E.O. (200 mg/kg b.wt.)			2.85* ± 0.16		1.78		25			2.50 ± 0.01	1.92 ± 0.01		1.30	
E.O. (400 mg/kg b.wt.)			3.62* ± 0.29		2.26		31			2.50 ± 0.02	1.91 ± 0.01		1.31	
EtOH ext. (200 mg/kg b.wt.)			3.28* ± 0.30		2.05		28			2.50 ± 0.02	1.90 ± 0.02		1.32	
EtOH ext. (400 mg/kg b.wt.)			10.58* ± 0.70		6.61		91			2.56 ± 0.01	1.96 ± 0.01		1.31	
	*n*-Hex. fr. (200 mg/kg b.wt.)		2.54* ± 0.28		1.59		22			2.54± 0.03	1.94 ± 0.03		1.31	
	*n*-Hex. fr. (400 mg/kg b.wt.)		2.78* ± 0.19		1.74		24			2.51 ± 0.01	1.91 ± 0.01		1.31	
	CH2Cl2 fr. (200 mg/kg b.wt.)		2.70* ± 0.18		1.69		23			2.51 ± 0.05	1.91 ± 0.01		1.31	
	CH2Cl2 fr. (400 mg/kg b.wt.)		2.82* ± 0.16		1.76		24			2.51 ± 0.01	1.91 ± 0.05		1.31	
Fractions of the 70%	EtOAcfr. (200 mg/kg b.wt.)		4.78* ± 0.29		2.99		41			2.53 ± 0.02	1.93 ± 0.02		1.31	
ethanol extract	EtOAcfr. (400 mg/kg b.wt.)		6.66 * ± 0.19		4.16		58			2.52 ± 0.01	1.93 ± 0.01		1.31	
	*n*-But. fr. (200 mg/kg b.wt.)		3.62* ± 0.11		2.26		31			2.50 ± 0.07	1.90 ± 0.01		1.32	
	*n*-But. fr. (400 mg/kg b.wt.)		6.30* ± 0.37		3.94		54			2.50 ± 0.01	1.91 ± 0.05		1.31	
	Rem. H2O (200 mg/kg b.wt.)		2.54* ± 0.30		1.59		22			2.52 ± 0.03	1.90 ± 0.01		1.33	
	Rem. H2O (400 mg/kg b.wt.)		3.49* ± 0.31		2.18		30			2.53 ± 0.02	1.89 ± 0.02		1.34	
Aq. ext. (200		mg/kg b.wt.)		3.24* ± 0.31		2.03		28	2.57 ± 0.03			1.98 ± 0.03		1.30	
Aq. ext. (400		mg/kg b.wt.)		5.26* ± 0.47		3.29		46	2.52 ± 0.02			1.92 ± 0.02		1.31	
		CH2Cl2 fr. (200 mg/kg b.wt.)		3.75* ± 0.29		2.34		32	2.49 ± 0.02			1.90 ± 0.08		1.31	
		CH2Cl2 fr. (400 mg/kg b.wt.)		4.80* ± 0.10		3.00		41	2.56 ± 0.01			1.89 ± 0.02		1.35	
		EtOAcfr. (200 mg/kg b.wt.)		3.25* ± 0.21		2.03		28	2.48 ± 0.01			1.96 ± 0.02		1.27	
Fractions of		EtOAcfr. (400 mg/kg b.wt.)		5.97* ± 0.27		3.73		52	2.58 ± 0.01			1.90 ± 0.01		1.36	
the aqueous extract		*n*-But. fr. (200 mg/kg b.wt.)		0.65 ± 0.05		0.41		6	2.55 ± 0.04			1.94 ± 0.01		1.31	
		*n*-But. fr. (400 mg/kg b.wt.)		0.85 ± 0.05		0.53		7	2.50 ± 0.01			1.90 ± 0.04		1.32	
		Rem. H2O (200 mg/kg b.wt.)		0.70 ± 0.08		0.44		6	2.55 ± 0.01			1.91 ± 0.01		1.34	
		Rem. H2O (400 mg/kg b.wt.)		0.90 ± 0.08		0.56		8	2.54 ± 0.01			1.91 ± 0.03		1.33	

Values are expressed as Mean ± SE.

EtOAcfr.: ethyl acetate fraction; EtOH ext.: 70 % ethanol extract; *n*-Hex. fr.: *n*-hexane fraction; Rem.: H2O, remaining water; SE: standard error.

* Statistically significant difference from zero time at *p *< 0.05. Aq.ext.: aqueous extract; *n*-But. fr.: *n*-butanol fraction; b.wt.: body weight; CH Cl fr.: methylene chloride fraction; E.O.: essential oil;

**Table 6:** Total phenolic, flavonoid and saponin contents in the different extracts and fractions

**Extract**	**TPC** * **± SE (GAE/100 g)**		**TFC** * **± SE (RE/100 g)**	**TSC** * **± SE (UAE/100 g)**
70% Ethanol ext. a.p.		5.97±0.001	15.85 ± 0.001	16.83 ± 0.001
	*n*-Hexane fr.	0.69 ± 0.001	2.25 ± 0.001	3.67 ± 0.001
	Methylene chloride fr.	0.78 ± 0.001	2.75 ± 0.005	4.83 ± 0.002
Fractions of the 70% ethanol extract of a.p.	Ethyl acetate fr. (EA)	9.38 ± 0.004	39.75 ± 0.005	9.00 ± 0.005
	*n*-Butanolfr.	6.86 ± 0.001	13.30 ± 0.007	30.00 ± 0.001
	Remaining water	1.38 ± 0.001	4.65 ± 0.001	27.00 ± 0.002
Aqueous ext. a.p.		5.63 ± 0.005	12.05 ± 0.004	31.50 ± 0.004
	Methylene chloride fr.	2.68 ± 0.001	9.50 ± 0.006	5.33 ± 0.001
Fractions of the aqueous extract of a.p.	Ethyl acetate fr.	6.86 ± 0.001	11.15 ± 0.001	15.17 ± 0.001
	*n*-Butanolfr.	0.71 ± 0.001	2.50 ± 0.001	36.17 ± 0.003
	Remaining water	0.69 ± 0.001	2.35 ± 0.001	31.67 ± 0.001

* average of three determinations; a.p.: aerial parts; ext.: extract; fr.: fraction; GAE:gallic acid equivalent; SE: standard error; TFC: total flavonoid content; TPC: total phenolic content; TSC: total saponin content.

Previous reports claimed that the flavonoids and saponins of the different *Solidago* species were responsible for the diuretic activity of the genus (7, 43 and 44). Others attributed the activity to their content of flavonoids and phenolics (6, 9 and 68). In our study, the highest diuretic activity of all tested samples was exhibited by the 70% ethanol extract. This high potency was presumably related to its high content of phenolics and flavonoids. This was further confirmed by the high diuretic activity of EA which possessed the highest phenolic and flavonoid contents among other fractions, the inactivity of the saponin rich *n*-butanol fraction of the aqueous extract, as well as the poor activity of the essential oil. This was in accordance with Apati *et al.* 2003 who stated that the flavonoids, especially quercetin and its derivatives showed a potential to inhibit the neutral endopeptidase enzyme, which is responsible for the interaction of the atrial natriuretic peptide through the excretion of the sodium ions (68). Moreover, the flavonoid fractions of some previously studied *Solidago *species showed diuretic activities, and the diuretic actions of several plant species were related to their flavonoid content (9, 69-71). The fractions of the 70% ethanol and aqueous extracts proved to be less active than the parent extracts, except for the ethyl acetate fraction of the aqueous extract, suggesting the existence of additive and/or synergistic effects in the parent extracts. The 70% ethanol extract of the flowering aerial parts of *S. canadensis* showed more powerful diuretic activity than the aquaretic drug Cystinol® (91% and 74% of furosemide activity, respectively) at the same dose level (400 mg/kg b.wt.) with a similar aquaretic property. It also showed a much higher diuretic activity when compared to the potassium sparing diuretic spironolactone (91% and 59% of furosemide activity, respectively) but without promoting the loss of sodium in urine. The tested 70% ethanol extract showed a comparable diuretic activity to the loop diuretic furosemide but without enhancing the loss of the electrolytes in urine (Na^+^ and K^+^). The increase in urine volume without loss of electrolytes showed that the tested samples were aquaretics, similar to the reference drug Cystinol®. 

Purification of the active ethyl acetate fraction (EA) yielded eleven compounds; thymidine **(1)**, isorhamnetin-3-*O*-*β*-ᴅ-glucopyranoside **(2)**, narcissin **(3),** kaempferol-3-*O*-(6”-*O*-acetyl)-*β*-ᴅ-glucopyranoside **(4)**, quercetin-3-*O*-(6”-*O*-acetyl)-*β*-ᴅ-glucopyranoside **(5)**, rutin **(6)**, kaempferol-3-*O*-*β*-ᴅ-apiofuranoside** (7)**, caffeic acid **(8)**, neochlorogenic acid **(9)**, quercetin **(10),** and kaempferol **(11)**. Compounds **1**_, _**2**_, _**4**_, _**5 **and **7 **were isolated for the first time from genus *Solidago*.

The spectral data of compound **1 **showed that the protons were assigned to the pyrimidine base thymine and deoxyribose sugar. The imino proton of the thymine base appeared at δ 11.27 ppm. The olefinic proton H-6 appeared at δ 7.70 ppm. The 3 protons of the methyl group at C-5 appeared as a singlet integrated as 3H at δ 1.78 ppm. The H-2′, H-3′, H-4′, and H-5′ protons of the deoxyribose sugar appeared at the region between 2.03 - 4.22 ppm. The anomeric proton of the deoxyribose sugar appeared as a triplet at δ 6.15 ppm. The ^13^C-NMR spectrum of compound **1 **revealed the presence of ten carbon atoms in the molecule. The ^13^C chemical shifts of a carbon at δ 12.72 indicated the presence of amethyl group attached to C-5. The assignment of the carbons of the pyrimidine base was determined using HMBC spectrum. Compound**1** was identified as thymidine. Compound** 7** showed the signals characteristic for a kaempferol nucleus and additional signals for a sugar moiety. The spectrum showed anomeric proton at 5.01 ppm as a doublet with J= 3.7 Hz characteristic for O-*β*-ᴅ-apiofuranose structure (63, 64, 72 and 73). ^13^C-NMR spectrum of compound **7** showed 18 carbon signals assigned to 20 carbons, 13 of which assigned to kaempferol (15 carbons) and 5 for apiose sugar. Signal of C-3 at 133.10 δ ppm was shifted upfield by 2 ppm relative to kaempferol aglycone (74). This confirmed the 3-glycosylation of kaempferol. Compound **7 **was identified as kaempferol-3-*O*-*β*-ᴅ-apiofuranoside. ^1^H-NMR spectrum of compound **9 **showed the characteristic signals for a caffeic acid molecule. Also the protons of a quinic acid moiety could be observed with a doublet at δ 1.73 (J = 12.9 Hz) assigned to H-6 ax, a doublet of doublet at δ 1.90 ppm (J = 13.8, 10.8 Hz) assigned to H-6 eq and a multiplet at 2.08 ppm integrated as two protons and assigned to H-2 _ax_ and H-2 _eq_. A broad singlet at δ 3.82 and a doublet at δ 3.87 ppm (J = 9 Hz) assigned to H-4 and H-5. The downfield shift of H-3 which appeared at δ 5.01 ppm indicated the acylation of the quinic acid by the caffeic acid at the OH on C-3 (75). The assignement of the protons of the quinic acid moiety was determined using ^1^H-^1^H COSY. Compound **9** was identified as neochlorogenic acid.

## Conclusion

Ethanol (70%) was the best solvent for extracting phenolic compounds from *Solidago canadensis *L., while water was the best solvent for the extraction of its saponins. The ethyl acetate fraction of the 70% ethanol extract (EA) of the flowering aerial parts possessed the highest total phenolic and flavonoid contents, as well as the highest diuretic activity, amongst all tested fractions. A strong correlation existed between the total phenolic and flavonoid contents and the investigated aquaretic activity of the different extracts and fractions. Thus, *S. canadensis* L. showed a pronounced aquaretic activity owing to its phenolic and flavonoid contents which was in accordance with Chodera *et al.*, 1991 (9). Eight flavonoids, 2 phenolic acids and 1 nucleoside were isolated and identified in the most active ethyl acetate fraction (EA). Four of them namely, isorhamnetin-3-*O*-*β*-ᴅ-glucopyranoside (**2**), kaempferol-3-*O*-(6”-*O*-acetyl)-*β*-ᴅ-glucopyranoside (**4**), quercetin-3-*O*-(6”-*O*-acetyl)-*β*-ᴅ-glucopyranoside (**5**) andkaempferol-3-*O*-*β*-ᴅ-apiofuranoside (**7**) were isolated for the first time from genus *Solidago*, while the other isolated compounds were previously reported (31, 37-40).
